# Transition metal atom (Ti, V, Mn, Fe, and Co) anchored silicene for hydrogen evolution reaction

**DOI:** 10.1039/c9ra04602j

**Published:** 2019-08-22

**Authors:** Yongxiu Sun, Aijian Huang, Zhiguo Wang

**Affiliations:** School of Electronic Science and Engineering, Center for Public Security Technology, University of Electronic Science and Technology of China Chengdu 610054 P. R. China 201811022517@std.uestc.edu.cn zgwang@uestc.edu.cn

## Abstract

Non-noble element catalysis for hydrogen evolution reaction (HER) is a promising pathway for mass hydrogen production through electrochemical water splitting. In this work, the catalytic performance of metal (alkali, alkali-earth, and transition metal) atoms anchored to silicene was investigated by density functional theory. Results showed that all the studied metal atoms are energetically favorably absorbed on the silicene with large binding energies. The pristine silicene is catalytically inert for HER, while the metal (Fe, V, Mn, Ti, Co, Ni, Be, and Cr) atom anchored silicene is catalytically active for HER with the calculated Gibbs free energies in the range between −0.09 and 0.18 eV, which is very close to the optimum value of 0.0 eV. These results suggested that the catalytic behavior of silicene can be effectively improved by metal adsorption. Such metal (Fe, V, Mn, Ti, Co, Ni, Be, and Cr) atom anchored silicenes can be used as potential catalysts for HER.

## Abstract 2

Ti, V, Mn, Fe, and Co anchored silicene as low-cost catalysts for hydrogen evolution reaction.

## Graphical abstract

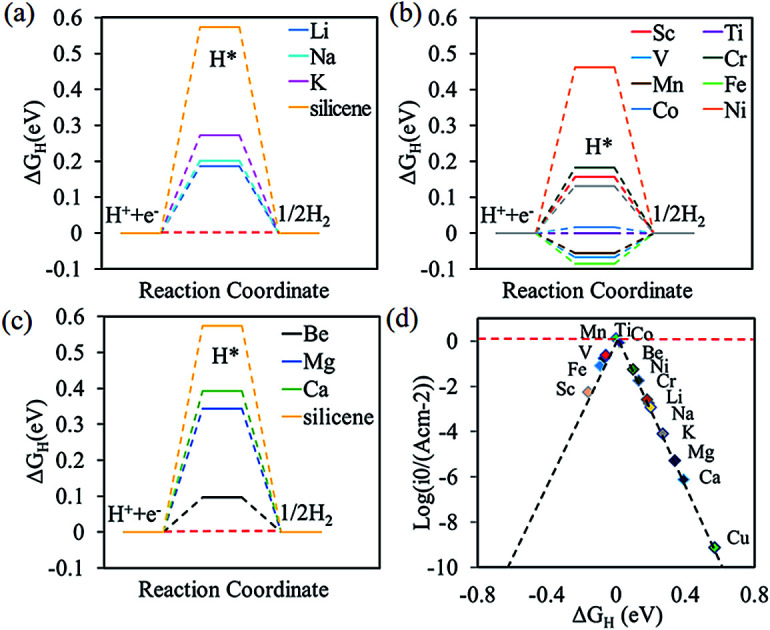

## 1. Introduction

The demand for energy is rapidly increasing with the growth of the world economy. Fossil fuels have been the main energy supply in the past few decades. In recent years, the energy crisis is becoming complicated due to the limited supply of fossil fuels and environmental concerns associated with carbon dioxide emission from the burning of fossil fuels.^1^ To meet the daunting energy demands in an environmentally sustainable way, the need of large-scale non-fossil fuel clean energy sources is urgent.^2^ Hydrogen, the simplest and relatively abundant element, has a high energy density by mass and is sustainable and eco-friendly as an alternative fuel.^3,4^ Among the various methods of hydrogen production, electrochemical water splitting is considered sustainable and useful for mass hydrogen production. Although the platinum-group metals work efficiently in the hydrogen production from water *via* hydrogen evolution reaction (HER), their scarcity and high cost hinder their widespread application. Developing efficient noble metal free electrocatalysts comparable to the platinum-group metals is urgent and remains a big challenge.^5^

Various non-noble metal electrocatalysts, including metal alloys, transition metal compounds, and carbonaceous nanomaterials,^6^ have been investigated for HER. Whereas all of them show some catalytic activity for HER, none of them are comparable with the platinum-group metal catalysts. Many kinds of schemes were investigated to further improve their catalytic performance. The electrochemical performance of metal alloys can be improved by increasing the density of the catalytically active sites for HER. For example, the HER performance of AuPd^7^ was enhanced though introducing porosity.^8^ Transition metal chalcogenides, such as MoS_2,_^9,10^ CoP,^11^ CoSe_2_,^12^ and WS_2_,^13^ show good catalytic performance, hence attracting much attention to be used as electrocatalysts for HER. However the catalytically active sites located at the edges site in either metallic or semiconducting transition metal sulfides,^14^ and in the trithio- or triseleno-phosphate compounds as well,^15^ thus leaving large surface site unavailable for HER. Doping has been used to increase the number of catalytically active sites, such as Se-doped MoS_2_,^16^ C-doped MoS_2_,^17^ Heterostructures, such as MoSe_2_/CoSe_2_,^18^ CoP/WS_2_,^19^ and MoSe_2_/NiSe,^20^ have also been evaluated as electrocatalysts for HER. Besides, many other approaches, such as decreasing the size,^8^ introducing defects,^21,22^ and controlling the phase transition^23–25^ in MoS_2_, have been proposed to increase the catalytically active sites.

Silicene, a single silicon atomic layer, has a graphene-like structure. The successful synthesis of silicene on metal surface using epitaxial growth method^26,27^ has given rise to a lot of attention due to its excellent electrochemical properties. The pristine silicene has a zero band gap,^28^ which can be tuned by in-plane strain and external vertical electrical field.^29,30^ The field-effect transistors based on the silicene has been fabricated by Li's group.^31^ The adsorption of some adatoms on silicene has been studied^32–36^ and the buckled silicene shows strong adsorption of the adatoms. The electronic properties of silicene can be modified by the adsorption of adatoms.^37^ The recent development of single-atom catalysis offers electrocatalytic mechanism with great potential,^38^ among which the isolated metal atom being anchored to two-dimensional materials has been well developed as catalyst for HER. The Pt atom confined into graphene showed higher catalytic activity than the conventional Pt nanoparticles.^39^ Pt and Co atoms adsorbed on g-C_3_N_4_ show good catalytic performance.^40^ The Pt,^41^ Pd,^42^ Zn,^43^ Ni,^44^ W,^45^ V, Fe, Co, Ni, Cu,^46^ and Se^47^ atoms adsorbed on MoS_2_ also show high catalytic performance for HER. This inspired us to study the strong binding of metal adatoms to silicene as catalyst for HER. Compared to other support materials, the silicene is also an atomic thick material with buckled layer structure, which results in large surface area.^48^ Although the silicene has a similar zero band-gap characteristic with graphene. It is expected silicene with high reactivity with adsorption of adatom due to the sp^3^-like buckled structure. Previous calculation has shown that many adatoms (such as Li, Na, K, Ca, Co, Ni, Pd and Pt) with stronger binding to silicene than to graphene.^36^ Importantly, Si is the second most abundant element on Earth, thus silicene is expected to be good support materials for single-atom catalysis.

In this paper, the catalytic activity of alkali, alkali-earth, transition metal atoms adsorbed on silicene for HER was investigated by using density functional theory (DFT). The calculation results showed that the pristine silicene is catalytically inert for HER due to the large positive value (0.57 eV) of Gibbs adsorption energy for hydrogen. However, transition metal atoms adsorbed on silicene, such as Ti and Co, shows an excellent catalytic activity for HER.

## 2. Computational details

The SIESTA code^49^ developed based on DFT was adopted for all the simulations. The electron-ion core interaction were described by using norm-conserving pseudopotentials, and the valence electron wave functions were expanded using a double-ζ basis set plus polarization function^50^ with an energy cut-off of 180 Ry. The generalized gradient approximation (GGA) of Perdew–Burke–Ernzerhof (GGA-PBE) functional was adopted to describe the electron exchange–correlation interaction.^50^ There are two atoms in the unit cell of silicene. The atomic positions and lattice constants were fully relaxed with geometry optimization by using conjugate gradient method until the force on each atom is less than 0.01 eV Å^−1^. The Brillouin zone (BZ) was sampled with a 20 × 20 × 1 *k*-point mesh^51^ for the unit cell of silicene. The plane of silicene is parallel to the *x*–*y* plane; a vacuum layer with a thickness of 30 Å along the *z*-direction was used to avoid periodic image interactions.

The electrocatalytic activity of alkali, alkali-earth, and transition metal adatoms adsorbed on silicene was evaluated by calculating the Gibbs free energy (Δ*G*_H_) of hydrogen adsorption. Δ*G*_H_ was calculated by the following equation:

1 Δ*G*_H_ = Δ*E*_H_ + Δ*E*_ZPE_ − *T*Δ*S*_H_

where Δ*E*_ZPE_ is the difference of zero-point energy between the adsorption state and gas phase, the value of Δ*E*_ZPE_ is calculated to be ∼0.16 eV.^52,53^ Δ*S*_H_ is the difference of entropy between the adsorption state of hydrogen and gas phase. At the standard temperature of 300 K and standard atmospheric pressure of 1 bar, the value of −*T*Δ*S*_H_ is calculated to be 0.20 eV.^23,52^ To sum up, the value of Δ*E*_ZPE_ − *T*Δ*S*_H_ is equal to 0.365 eV. Δ*E*_H_ was calculated by the eqn (2):2
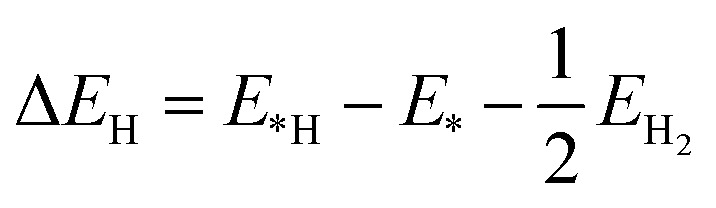
where E_*H_ and E_*_ are the total energies adatom adsorbed silicene with and without hydrogen adsorption, respectively. *E*_H_2__ is the total energy of hydrogen molecule in the gas phase.^54^

The basis set superposition error (BSSE) induced by the artificial shortening of distances and strengthening of the interactions was corrected by applying the counterpoise corrections using ‘ghost’ atoms.^55,56^

## 3. Result and discussion

**Fig. 1:**
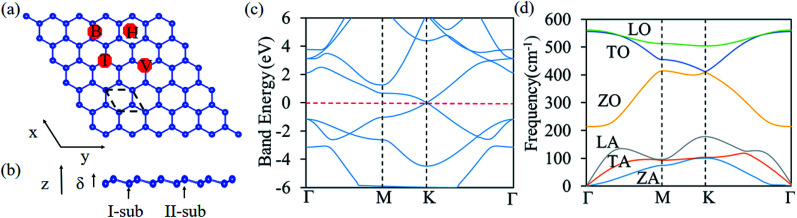
(a) Top and (b) side view of silicene, and the top and bottom atoms are labeled as I- and II-sublattice. The rhombus denotes the primitive unit cell of silicene. (c) Band structure and (d) phonon dispersion of silicene.

Silicene has a hexagonal atomic arrangement, with the top and side views of silicene is shown in Fig. 1a and b, respectively. The dashed rhombus denotes the unit cell of silicene. The silicene has a wrinkled surface, which consists of I- and II-sub-structure atoms.^57^ Using first principle calculation, the lattice constant of silicene is calculated to be 3.86 Å with the bond length of 2.28 Å for Si–Si bond. The buckling distance (*δ*) is 0.51 Å. The angle of ∠Si–Si–Si is 115°, which is in good agreement with the previous work.^58,59^ The angle between Si–Si bond and the *z* axis is 122°. The band structure of silicene is shown in Fig. 1c. Silicene is a zero band-gap material with both the valence band maximum (VBM) and conduction band minimum (CBM) located at *k* point, both crossing the linear dispersion with the Fermi level.^60^

The phonon dispersion of silicene was calculated by using the frozen phonon method^50^ with a 6 × 6 supercell. Phonon dispersion of silicene along the high-symmetry direction is shown in Fig. 1d. There are two atoms in the unit cell, one is for I-sublattice and the other one for the II-sublattice, so there are six branches of phonon spectrum consisting of longitudinal acoustic (LA), transverse acoustic (TA), flexural out-of-plane acoustic (ZA), longitudinal optical (LO), transverse optical (TO), and flexural out-of-plane optical (ZO), which agrees with the previous results.^61,62^ The ZA branch near the *Γ* point shows a quadratic trend, which is a typical characteristic of two-dimensional materials and can be explained by the macroscopic elastic theory.^61,62^ There is no imaginary frequency for the silicene, which indicates that the silicene is dynamically stable.

The strong binding of adatoms to a two-dimensional is beneficial to maintaining a long cycle life for catalytic performance. The binding between the metal adatom and silicene was evaluated by using the eqn (3):

3 *E*_ads_ = *E*_adatom_ + *E*_silicene_ − *E*_adatom@silicene_

where *E*_adatom@silicene_ and *E*_silicene_ are the total energies of silicene with and without metal adatom adsorption, respectively. *E*_adatom_ is the energy of an isolated metal adatom. The lager the adsorption energy is, the stronger binding between adatom and silicene is. There are four possible symmetry adsorption sites for adatoms on the surface of silicene (as shown in Fig. 1a and b), which are the top of upper silicon atom (T), the valley site, *i.e.* the top of bottom silicon atom (V), the bridge site of Si–Si bond (B), and the center of the hexagon (H). The calculated binding energies are listed in Table 1.

**Table 1:** Calculated binding energy (eV) of alkali, alkali-earth, and transition metal adatoms anchored to silicene. The catalytic active sites for HER with the Gibbs free energies for hydrogen adsorption. And adsorption energy (eV) of hydrogen molecules on pristine, alkali, alkali-earth, and transition metal atoms anchored silicene

	Binding energy (eV)					HER		Adsorption energy(eV)	
	T	V	H	B	Optimum site	Adsorption site	Δ*G*_H_ (eV)	Adsorption site	*E*_ads_(H_2_) (eV)
Li	2.16	2.34	2.65	2.34	H	T	0.19	T	0.01
Na	1.85	1.99	2.22	1.99	H	T	0.20	T	0.00
K	2.25	2.06	2.25	2.06	H	T	0.27	T	−0.06
Be	0.61	1.54	1.24	1.53	V	Tad	0.10	Tad	−0.02
Mg	0.63	1.16	1.04	1.16	V	Tad	0.34	Tad	0.00
Ca	1.37	1.37	1.29	1.36	V	T	0.39	Tad	0.16
Sc	4.54	4.12	4.31	4.54	B	T	−0.16	T	−0.31
Ti	5.20	5.44	5.70	5.43	H	Tad	−0.01	H	−0.02
V	6.32	6.16	6.32	6.16	H	Tad	−0.07	T	0.00
Cr	6.37	6.54	6.61	6.54	H	T	0.18	T	0.00
Mn	6.45	6.56	6.87	6.56	H	T	−0.06	T	0.00
Fe	6.47	6.58	7.17	6.58	H	T	−0.09	T	−0.01
Co	7.61	7.79	8.47	7.79	H	Tad	0.02	T	−0.01
Ni	6.97	7.21	7.97	7.21	H	T	0.13	T	0.00
Cu	4.33	4.51	5.02	4.51	H	T	0.57	T	0.00
Pristine	—	—	—	—	—	T	0.57	T	0.00

**Fig. 2:**
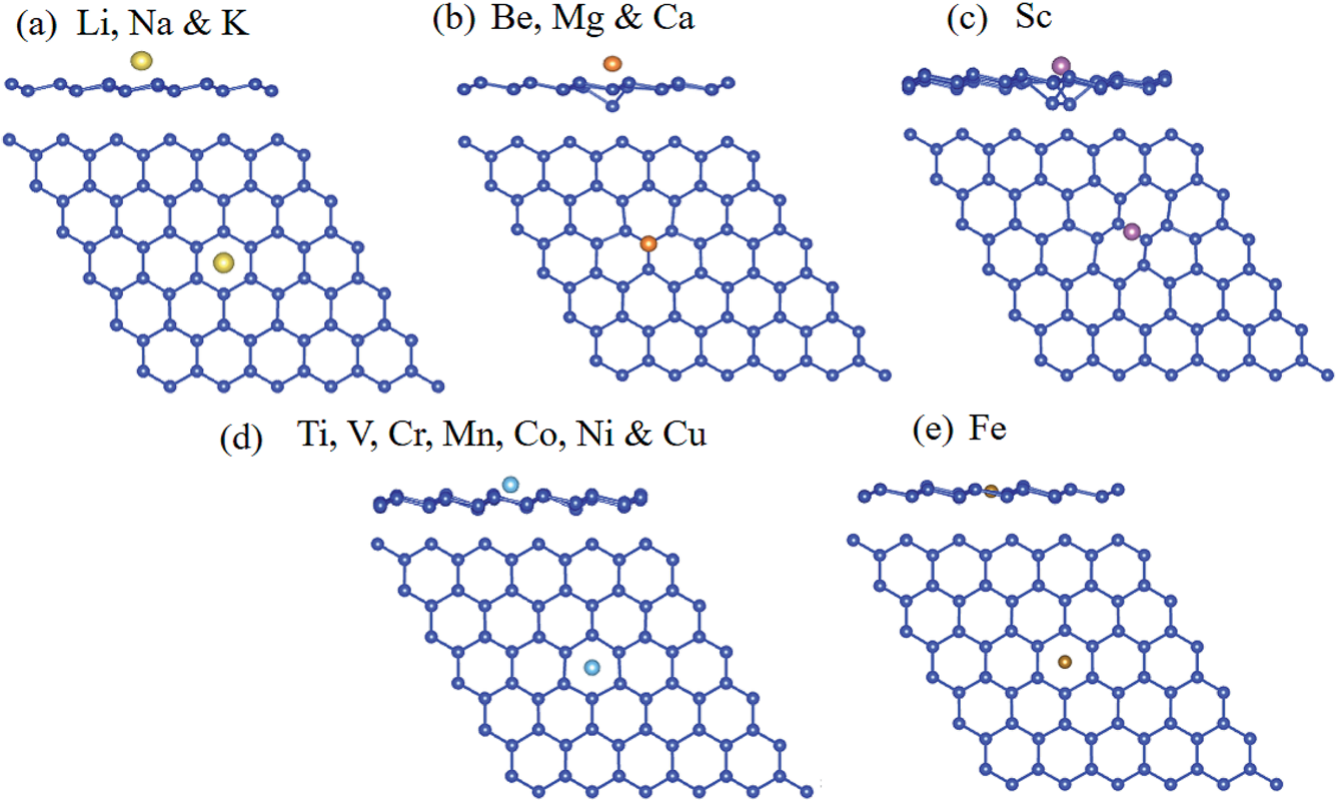
Top and side view for bonding geometry structures for (a) alkali, (b) alkali-earth, (c) Sc, (d) transition metal (except Fe atom) and (e) Fe atoms on silicene.

For the alkali adatoms (Li, Na, and K) adsorption on the silicene, all of them are strongly bound to the H site. The binding energies are 2.65, 2.22, and 2.25 eV for Li, Na, and K, respectively. The geometry structure of alkali atoms absorbed on silicene is shown in Fig. 2a. Similar to the alkali atom absorbed on graphene,^63^ as the alkali adatoms are absorbed on the silicene, the valence electrons of alkali atoms transfer to the silicene, resulting in strong binding between them. The alkali-earth atoms show different energy preferable adsorption sites compared with alkali atoms, favoring to be absorbed at the V site, as shown in Fig. 2b. The binding energies are 1.54, 1.16, and 1.37 eV for Be, Mg, and Ca adatoms, respectively. From Table 1, it can be seen that except Sc, all the studied transition metal adatoms are strongly bounded to the H site of silicene. The atomic configurations are shown in Fig. 2c. The bridge site is energetically favorable one for Sc adsorption. The binding energies are in the range between 4.54 and 8.47 eV, much larger than the alkali and alkali-earth atoms. It is interesting to find that the Fe atom is absorbed at the center of the hexagon in the silicene plane. The transition metal adatoms (Ti, V, Cr, Mn, Fe, Co, Ni, and Cu) are strongly bounded to the H site of silicene and form covalent bonds with the nearest Si atoms. Among them, the Mn, Fe, Co, Ni and Cu atoms have smallest atomic radius. The bond lengths of Mn–Si, Co–Si, Ni–Si and Cu–Si bonds are 2.35, 2.60, 2.61, and 2.70 Å, respectively. Whereas the bond-length of six Fe–Si bond is about 2.32 Å, which is close to Si–Si bond length (2.28 Å), results in the Fe atoms absorbed at the center of the hexagon in the silicene plane. These results are in agreement with the previous work.^36^ The strong binding energies are beneficial for them as catalyst for HER.

**Fig. 3:**
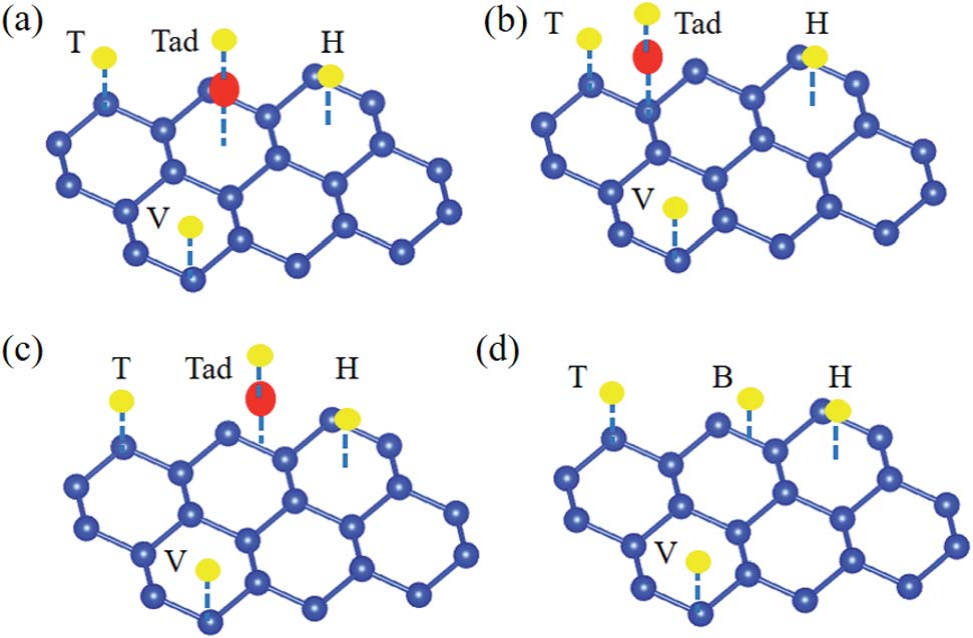
The possible adsorption sites of H atom on adatom@silicene with the metal atom absorbed at the (a) H, (b) V, (c) B sites and (d) pristine silicene.

After identifying the favorable adsorption sites for adatoms on the silicene, the catalytic performance for HER of the alkali, alkali-earth, and transition metal atoms anchored on the silicene was investigated by the calculation of the Gibbs free energy for hydrogen adsorption. For the pristine silicene, the H is preferable to be absorbed at T site, given the large Δ*G*_H_ of 0.57 eV, which is much smaller that H absorbed on graphene (1.80 eV).^64,65^ This result is also caused by the high reactivity of the sp^3^-like buckled structure compared to the sp^2^-hybridization in graphene.^37^ The large positive value means that the hydrogen is difficult to bind to the silicene, indicating the pristine silicene is catalytically inert for HER. The silicene with metal adatom anchored (adatom@silicene) can be classified into three types according to their adsorption sites, *i.e.* the H type (Li, Na, K, Ti, V, Cr, Mn, Co, Ni, Cu, and Fe), V type (Be, Mg, and Ca) and the B type (Sc). The possible adsorption sites for hydrogen on the three types of adatom@silicene systems are shown in Fig. 3a–c. Four possible adsorption sites are considered, *i.e.* the top of the adatom (Tad), the top of upper silicon atom (T), the valley site (V), and the center of the hexagon (H). The energetically favorable adsorption sites for hydrogen on adatom@silicene system along with the calculated Δ*G*_H_ are listed in Table 1. A large positive or negative value of Δ*G*_H_ indicates that hydrogen adsorption on the catalyst is too strong or too weak, both of them are not beneficial for the HER.^66^

**Fig. 4:**
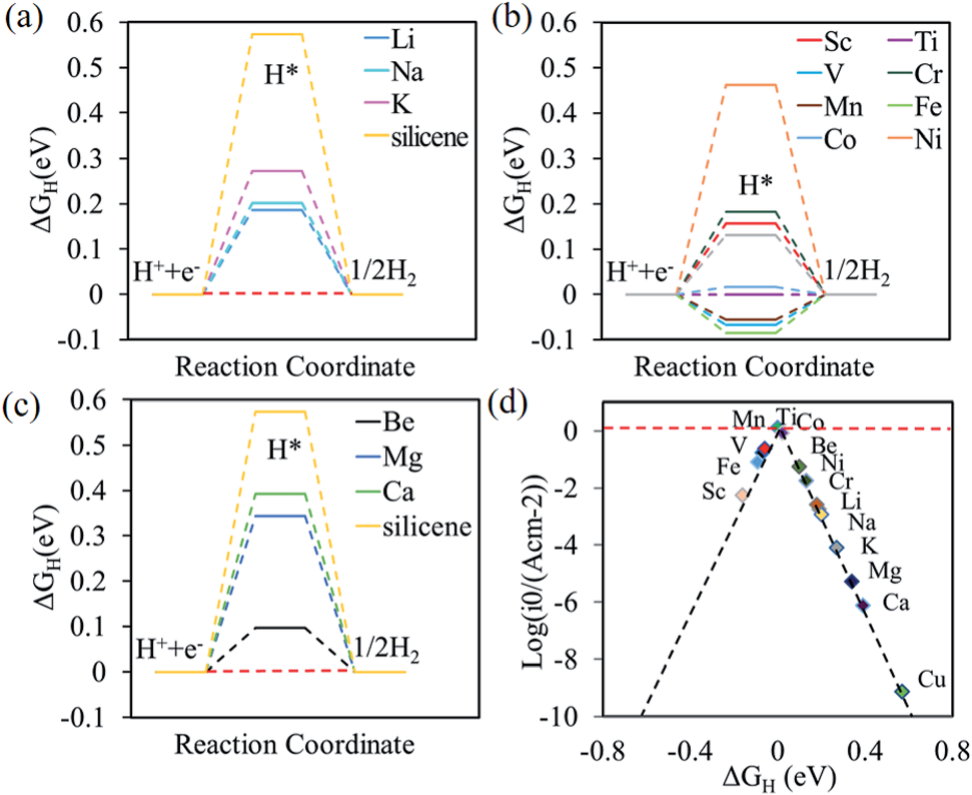
Gibbs free energy diagram of HER at the equilibrium potential for (a) alkali, (b) alkali-earth, and (c) transition metal atoms anchored silicene, (d) volcano curve of exchange current as a function of the Gibbs free energy.

The free energy diagrams of alkali, alkali-earth, and transition metal atoms anchored to silicene for HER are shown in Fig. 4a, b, and c, respectively. The values of Δ*G*_H_ decrease from 0.57 to 0.19, 0.20, and 0.27 eV for the Li, Na, and K@silicene, respectively, indicating that the catalytic performance can be improved by the alkali atom adsorption. However, these values of Δ*G*_H_ are still larger than the optimum value of 0.0 eV, thus the alkali atom anchored to silicene is not a good catalyst for HER. The value of Δ*G*_H_ is decreased for H adsorbed on alkali-earth atoms anchored to silicene. The values of Δ*G*_H_ is 0.10 eV for hydrogen adsorbed on Be@silicene, indicating Be@silicene is a potential catalyst for HER. Most of the transition metal atoms anchored to silicene show good catalytic behavior for HER due to the small absolute value of |Δ*G*_H_|, such as 0.02, −0.01, −0.06 eV for hydrogen adsorbed on Co@silicene, Ti@silicene, and Mn@silicene, respectively.

The catalytic performance of HER is mainly determined by the adsorption ability of reaction intermediate (H*), which can be evaluated by the exchange current density as a function of Gibbs free energy of hydrogen adsorption in the shape of a volcano plot.^67^ Based on the assumption of Norskov,^68^ the theoretical exchange current *i*_0_ was calculated by using eqn (4) and (5) for Δ*G*_H_ >0 and Δ*G*_H_ < 0, respectively, at pH = 0,4
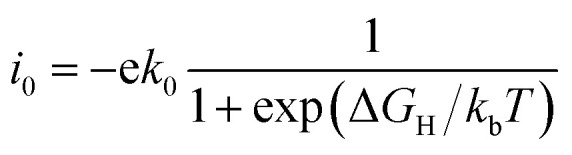
5
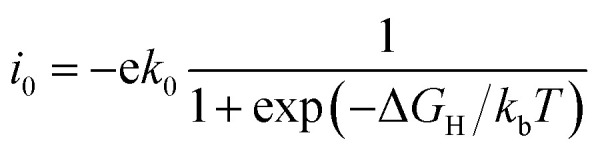
where *k*_0_ is the reaction rate constant, *k*_b_ is the Boltzmann constant, and *T* is the temperature. The volcano curve for the alkali, alkali-earth, and transition metal atoms anchored to silicene is shown in Fig. 4d. The catalytic activity is related to the positions of *i*_0_ and Δ*G*_H_, the closer the position of these values to the peak, the better the catalyst is. As can be seen from Fig. 4d, the values of Δ*G*_H_ for Sc, Fe, Mn and V anchored to silicene are in the left side of the volcano curve. The value of Δ*G*_H_ for Sc@silicene is far away from the volcano peak, indicating the hydrogen is tightly bound to Sc@silicene. While other adatoms anchored to silicene are in the left side of the volcano curve, the values of Δ*G*_H_ for Li, Na, K, Mg, Ca, and Cu@silicene are with larger distance to the peak, indicating that the hydrogen is difficult to be bound to the catalyst. The Fe, V, Mn, Ti, Co, Ni, Be, and Cr are very close to the peak with maximum exchange current, indicating they are catalytically active for HER as they are anchored to the silicene.

As a good catalyst, the molecular hydrogen should be released from the catalyst site easily. The adsorption energy (*E*_ads_(H_2_)) of the molecular hydrogen on pristine, alkali, alkali-earth, and transition metal atoms anchored silicene was calculated using the eqn (6):

6 *E*_ads_(H_2_) = *E*_H_2_@system_ − *E*_H_2__ − *E*_system_

where *E*_H_2_@system_ and *E*_system_ are the total energies of pristine, alkali, alkali-earth, and transition metal atoms anchored silicene with and without H_2_ adsorption, respectively. *E*_H_2__ is the energy of hydrogen molecule. According to the equation, the larger the adsorption energy is, the easier for the releasing of H_2_ is.^69^ All the four possible symmetry adsorption sites as H absorbed on the surface of adatom@silicene (as shown in Fig. 1a and b) were considered for H_2_ adsorption. The calculated adsorption energies and energy favorable sites are listed in Table 1. The adsorption energies are −0.01, 0.00, 0.00, −0.02, −0.01, 0.00, −0.02, and 0.00 for the Fe, V, Mn, Ti, Co, Ni, Be, and Cr anchored silicene, respectively, indicating that these systems are highly active for the releasing H_2_ gas.

## 4. Conclusion

In conclusion, the adsorption of alkali, alkali-earth, and transition metal adatoms on silicene as well as the catalytic performance of adatom anchored silicene for HER were investigated using DFT calculation. All the metal atoms are strongly bound to the silicene with large binding energy. The alkali adatoms (Li, Na, and K) are strongly bound to the H site, the alkali-earth atoms favors to be absorbed at the V site, the transition metal atoms except for Sc are strongly bounded to the H site while Sc is bound to the B site. The metal atoms adsorption can enhance the catalytic performance of silicene. The values of Δ*G*_H_ are −0.01, −0.07, −0.06, −0.09 and 0.02 eV for Ti, V, Mn, Fe, and Co atoms anchored to silicene, respectively. These results suggest that the metal adatom (Ti, V, Mn, Fe, and Co) anchored to silicene offer promising low-cost catalysts for HER.

## Conflicts of interest

There are no conflicts to declare.

## Supplementary Material
